# Global malaria eradication and the importance of *Plasmodium falciparum* epidemiology in Africa

**DOI:** 10.1186/s12916-014-0254-7

**Published:** 2015-02-03

**Authors:** Robert W Snow

**Affiliations:** Spatial Health Metrics Group, Department of Public Health Research, KEMRI-Welcome Trust Research Program, Nairobi, Kenya; Centre for Tropical Medicine and Global Health, Nuffield Department of Clinical Medicine, University of Oxford, CCVTM, Oxford, UK

**Keywords:** Africa, Eradication, Malaria, *Plasmodium falciparum*

## Abstract

The global agenda for malaria has, once again, embraced the possibility of eradication. As history has shown, there will be no single magic bullet that can be applied to every epidemiological setting. Africa has a diverse malaria ecology, lending itself to some of the highest disease burden areas of the world and a wide range of clinical epidemiological patterns making control with our current tools challenging. This commentary highlights why the epidemiology of *Plasmodium falciparum* malaria in Africa should not be forgotten when planning an eradication strategy, and why forgetting Africa will, once again, be the single largest threat to any hope for global eradication.

## Background

Malaria is a mosquito borne disease which, in humans, is caused by five protozoa: *Plasmodium falciparum*, *P. vivax*, *P. malariae*, related sibling species of *P. ovale*, and *P. knowlesi. P. vivax* is the most cosmopolitan of the human malarias, reaching historical latitudinal extremes of 64° north and 32° south [1]. The public health burden posed by *P. vivax* is no longer regarded as benign, causing severe morbidity and death [2]. Nevertheless, *P. falciparum* remains the single most important threat to public health at a global scale, accounting for more than 90% of the world’s malaria mortality.

Forty thousand years on, *P. falciparum* remains entrenched in Africa, largely as a result of optimal environmental conditions for the world’s most efficient Anopheline mosquito vectors, amid sustained poverty. We remain unacceptably ignorant of the full extent of the public health burden posed by this parasite; however, it is clear that, over time, the mortality effects of *P. falciparum* have been significant, serving as a potent selective force on the human genome to confer red cell and hemoglobin genetic advantages against disease and death [3]. It is reasonable to assume that Africa has contributed most to the global malaria burden for millennia.

### Advances and challenges with malaria control

Incredible progress was made following the Second World War, with the discoveries of DDT and chloroquine, shrinking the global extents of both *P. vivax* and *P. falciparum*, benefiting large parts of the Americas, Europe, and Asia. However, the elimination ambition in sub-Saharan Africa came to an abrupt end by the early 1960s when it was recognized that interrupting transmission with indoor residual house-spraying and/or mass drug administration was almost impossible [4].

Revisiting this early literature has important implications for the control of malaria today. First, targeting adult vectors and the parasite was far more successful than targeting the vector alone [5]; second, despite not always being able to interrupt the incidence of new infections, the disease burden and mortality plummeted to very low levels when there was complete intervention coverage; finally when these ‘experiments’ came to an end, clinical and fatal events returned quickly to pre-intervention levels and in some cases with worsened consequences [6].

Malaria across Africa reached epidemic proportions in the 1990s [7], leading to the launch of a new global strategy in 2000 with Africa center stage. During the Global Malaria Eradication campaign of the 1950s, building a national understanding of the epidemiology of transmission, cartography of risk, endemicity, and vector species distributions was vital. In addition, detailed pilot investigations molded decisions on whether elimination was feasible, what was required, and where within a country it might achieved. This level of epidemiological intelligence was absent at the launch of the Roll Back Malaria campaign in 2000.

### Epidemiological intelligence

**Figure 1:**
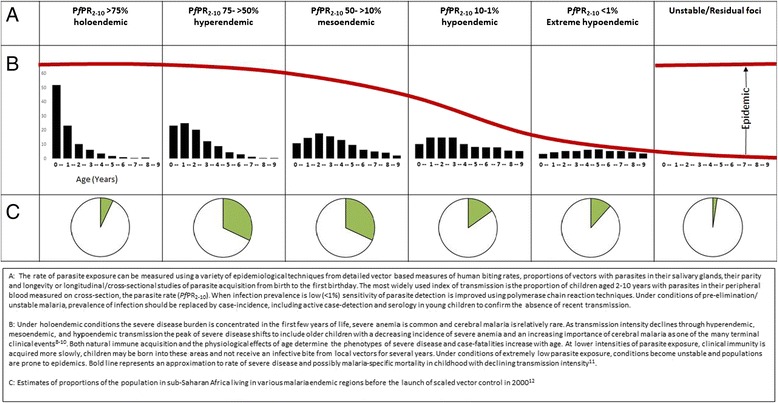
**Conceptual framework of the clinical epidemiology of** ***Plasmodium falciparum*** **under declining parasite transmission intensity in Africa.**

The clinical epidemiology of *P. falciparum* is complex and interest in unraveling its mysteries goes back over 80 years. What we do know is that repeated parasite exposure leads to the acquisition of a clinical immunity that first protects against the severe consequences of infection, immunity develops more slowly to the milder forms of the disease and much more slowly by regulating the intensity of blood stage infection. We do not know how many new parasite exposures are required to invoke a functional clinical immune response, but in areas where multiple new infections are experienced quickly from birth, immunity is acquired faster than in an area where the intensity of parasite transmission is much lower. This remains one of the fundamental conceptual frameworks of malaria disease epidemiology that governs the age and clinical patterns of health outcomes, rates of morbidity and mortality, and the varying mixes of control options available on an elimination pathway (Figure 1).

There is evidence [8-12] to suggest that despite a changing pathogenesis and age pattern of disease, the overall rate of severe, life-threatening disease in childhood remains stable for a large part of the transmission curve (bold line in Figure 1) and only when conditions within the mesoendemic range are reached do the rates of disease begin to significantly decline. As transmission declines further, the risk of infection becomes more directly related to the chances of becoming sick and developing severe complications until a state is reached where infection risks and disease outcomes are both rare. Lacking functional immunity, the consequences of any new infection for an individual become increasingly severe. These unstable conditions become very susceptible to even the smallest of perturbations in climate, ecology, population movement, and intervention efficacy (drug and insecticide resistance) or coverage.

Throughout the transmission spectrum, malaria adheres to some basic infectious disease principles: some people are more susceptible to a poor disease outcome than others, some are bitten more frequently than others, and some are more infectious than others [13]. This heterogeneity becomes increasingly relevant to control with declining intensity of transmission to the point below 1% *Pf*PR _2–10_, where foci of risks emerge. When elimination plans are activated, these sinks of transmission become the target of intervention.

After a decade of Roll Back Malaria in Africa, almost 184 million Africans still live under conditions of hyper-holoendemic transmission [12], despite impressive coverage of insecticide-treated nets (ITNs) since 2006. Mathematical theory suggests that ITNs alone might not reduce parasite exposure enough in the highest endemicity classes, even when deployed to protect over 80% of the population and used every night [14,15]. The same theory suggests that ITNs with similar coverage and use will transition communities from a natural mesoendemic range to the lower end of the hypoendemic range within 5 to 8 years. There is an increasing body of evidence to suggest we are witnessing intractable disease risks across the intense transmission belt of middle Africa; transmission drops moderately, age patterns of disease re-align, but overall disease incidence remains the same or rises [16-20].

What is imperative is that sick patients are diagnosed properly and managed quickly and with the right drugs. This is true across the entire transmission spectrum. The vectors and the parasite are not going anywhere soon and there is a school of thought that some of the greatest benefits will come when Africa is lifted out of poverty. Urbanization will have an impact on vector abundance and species composition. Improved health systems will increase access to therapeutics and prophylactics targeting the parasite as well as the ability to track residual foci of risk. All would benefit from a growing economy. It is no coincidence that malaria was finally eliminated from southern European countries and the United States at times when economies were exponentially expanding.

## Conclusions

As we pursue an agenda to shrink the world malaria map, Africa remains the focus of greatest disease burden and cannot be forgotten. Single approaches based on human-vector contact might be inadequate in high transmission areas. In the absence of a vaccine, how do we tackle the intractable heartland of high transmission? Where ITNs have been scaled up and communities have transitioned to hypoendemic states, the remaining foci will serve to catalyze epidemics if intervention coverage is scaled down. How do we simultaneously target vectors and parasites to maximize impact on transmission? What cost effective interventions and how should these be deployed in traditionally low, stable, and unstable margins of Africa? Answers to these questions must be guided by a better epidemiological framework and ample data. Ignorance of the epidemiological diversity that characterizes Africa and the challenges it poses to sustained control and elimination will be the single largest threat to the global eradication agenda.

## Abbreviation

ITNs: Insecticide-treated nets
